# Phenotypic Diversity and Outcomes in Pediatric NMDA Receptor Encephalitis: A 15‐Year Retrospective Study from the Largest Children's Hospital in the United States

**DOI:** 10.1002/advs.202520313

**Published:** 2026-03-28

**Authors:** Alexander J. Sandweiss, Timothy Erickson, Yike Jiang, Varun Kannan, Jonathan M. Yarimi, Kristen S. Fisher, Nikita Shukla, Tim Lotze, Eyal Muscal, Kristy O. Murray

**Affiliations:** ^1^ Department of Pediatrics Division of Pediatric Neurology and Developmental Neuroscience Baylor College of Medicine and Texas Children's Hospital Houston Texas USA; ^2^ Department of Pediatrics Section of Pediatric Tropical Medicine Center for Human Immunobiology Baylor College of Medicine and Texas Children's Hospital Houston Texas USA; ^3^ Laboratories for Emerging and Tropical Diseases School of Public Health Texas A&M University College Station Texas USA; ^4^ Department of Pediatrics Division of Pediatric Rheumatology Duke University School of Medicine Durham North Carolina USA; ^5^ Department of Pediatrics Division of Pediatric Neurology Emory University School of Medicine and Children's Healthcare of Atlanta Atlanta Georgia USA; ^6^ Memorial Division of Pediatric Neurology Joe DiMaggio Children's Hospital Hollywood Florida USA; ^7^ Department of Pediatrics Division of Innovation and Research Emory University School of Medicine and Children's Healthcare of Atlanta Atlanta Georgia USA

**Keywords:** autoimmune encephalitis, neuroimmunology, neuroinflammation, pediatric neurology

## Abstract

Anti‐NMDAR encephalitis (NMDARE) is an autoantibody‐mediated disorder characterized by seizures, movement disorders, neurocognitive deficits, and psychosis, but the complete phenotypic heterogeneity, and outcomes are incompletely understood in children. This single‐center retrospective analysis of NMDARE at the largest pediatric hospital in the United States between 2009 and 2024 screened 115 patients diagnosed with NMDARE. 103 had sufficient clinical data available for analyses. Two‐thirds were Hispanic, disproportionate to the Houston metro area demographics, and Hispanic patients had a higher CSF white cell count and antibody titer. Approximately one‐half of the patients with idiopathic NMDARE presented with a focal cortical phenotype that localized to the perisylvian region as initial symptomology, which we describe as a “perisylvian phenotype.” Patients with teratomas had more severe early symptoms, earlier lumbar punctures, higher CSF white cell counts, earlier treatment, and longer hospital durations than HSVE and idiopathic patients. CSF antibody titers directly correlated to hospital length of stay and mRS at presentation through 12‐month follow up, and normal routine CSF studies and brain MRI delayed initiation of first‐line immunotherapy. These novel and corroborating observations serve as the foundation for future studies on early focal neurological deficits (i.e., perisylvian phenotype) that must be addressed by clinicians to prevent delay in care.

## Graphical abstract

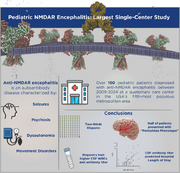

## 1 Introduction

Anti‐N‐methyl‐D‐aspartate receptor encephalitis (NMDARE) is an autoantibody‐mediated disorder characterized by seizures, movement disorders, neurocognitive deficits, psychosis, and may lead to lifelong neuropsychiatric sequelae even with early immunomodulation [1, 2]. While much of the focus in encephalitis has been on defining the natural history in adults, there have been a paucity of large pediatric studies [3, 4, 5, 6, 7]. Further, the literature on pediatric NMDARE (pNMDARE) is mostly derived from generally homogenous populations [8] that may narrow the potential presenting symptoms and outcomes. In 2012, the California Encephalitis Project (CEP) described the major clinical differences between NMDARE and infectious encephalitis from herpes simplex virus (HSV) and West Nile virus (WNV), and found the NMDARE phenotypes were considerably skewed toward movement disorders and psychosis [9]. This dichotomy has largely remained true in the literature over the past decade, but further delineation of symptomology has not been stratified beyond movement disorders, psychosis, seizures, and perhaps non‐specific sensory and motor changes. Moreover, NMDARE is typically described broadly as a “CNS inflammatory disorder” without further localization of specifically affected brain nuclei, which is likely dependent on the topographic NMDA receptor expression profile [10, 11].

Most reports on pNMDARE come from individual case reports, while the largest clinical pNMDARE studies to date are typically multicenter‐design with heterogeneity in practice patterns, or single‐center design but considerably smaller sample sizes [12 ^–^ 17]. We sought to characterize the demographics (including race, ethnicity, sex, age and language spoken), presenting symptoms, clinical markers, practice patterns, and outcomes for a contemporary pNMDARE cohort with standardized care algorithms from the United States’ largest children's hospital in the country's fifth‐most populous Metropolitan Statistical Area and a catchment area spanning nine counties [18, 19].

## 2 Experimental Section

### 2.1 Study Design and Patient Population

This is a single‐center retrospective analysis of pNMDARE at Texas Children's Hospital in Houston, TX, USA, between 2009 and 2024, where NMDARE international consensus guidelines have been implemented since 2009. Guidelines evolved over the duration of our study. Therefore, patients were identified on our inpatient floor, ICU, ER, or in the outpatient rheumatology or neurology clinics to have pNMDARE, as determined by:

‐ Between 2009 and 2020: presenting with subacute onset of neurological or psychiatric symptoms such as seizures, focal neurological deficits, or psychosis, with the presence of anti‐NR_1_ antibodies in either CSF, serum, or both, as described by Dalmau et al. in 2008 [1]
‐ Between 2020 and 2024: meeting the “Cellucci Criteria [20]” (an adaptation of the “Graus Criteria” applied to children) of presenting with subacute onset (< 3 months) new focal neurologic deficits, cognitive difficulties, movement abnormalities, psychiatric symptoms, and/or seizures with the presence of anti‐NR_1_ antibodies in CSF, serum, or both.

We present the demographics and etiologies of the total screened cohort that met the inclusion criteria first, then we apply exclusion criteria in order to focus our primary analyses on a cohort with the most complete data available and eliminate extreme outliers in disease course. Subjects were excluded from primary analyses if:

‐ Data were not available regarding: date of symptom onset, date of initial presentation to healthcare, date of initial anti‐NR_1_ antibody analysis
‐ First‐line therapy such as either high‐dose intravenous methylprednisolone (IVMP) or intravenous immunoglobulin (IVIG) was not administered within 100 days of symptom onset

### 2.2 Extracted Variables

CSF and serum studies were evaluated via cell‐based assay (CBA, as previously described in ref. [1]) by either Mayo Clinic Labs (Rochester, MN), ARUP Labs (Salt Lake City, UT) or both, and less commonly evaluated by Athena Diagnostics (Marlborough, MA) or by the lab of Dr. Dalmau early in the history of NMDARE's discovery. The sample was considered positive for the presence of anti‐NR_1_ antibodies if it demonstrated the requisite immunofluorescence on transfected HEK293 cells at a dilution of at least 1:1 in CSF and at a dilution of at least 1:10 in serum. Demographics collected included: self‐identified race and ethnicity, age, sex, and language spoken, and variables necessary to determine Area Deprivation Index (ADI) as a surrogate for socioeconomic disadvantage [21, 22]. Additionally, clinical information, labs, imaging, and EEGs were obtained from the electronic medical records of each patient. Encounters were defined as in‐person healthcare encounters with physicians or equivalent, most commonly the emergency room or outpatient primary care physician. CSF pleocytosis was defined as > 5 cells/uL and normal CSF protein defined as between 15 and 60 mg/dL [23]; analysis involving CSF did not include any neonates (for whom pleocytosis and protein may be defined differently). While limited in complete applicability across the spectrum of patients with NMDARE, particularly in children, the anti‐NMDAR Encephalitis 1‐Year Functional Status (NEOS) score was calculated for each patient with the requisite data available as this tool is the most widely used prognostic modality to predict poor functional outcomes [24].

Data were presented as percent positive for dichotomous variables and as average ± standard error of the mean for continuous numerical variables. Latencies to events are described as the median and range, and antibody (Ab) titers were log‐transformed using the formula *y* = log_2_(*x*)+1 such that the transformation reflects the “dilution factor” of the titer (i.e., 1:1 = 1, 1:2 = 2, 1:4 = 3, 1:8 = 4, etc) [25 ^,^ 26].

Electroencephalogram (EEG) was defined as ‘unremarkable’ if there were no abnormalities for age and a posterior dominant rhythm (PDR) was present, generalized slowing or generalized beta activity with a PDR present, and ‘abnormal’ if there were any focal features (such as slowing, beta, rhythmic delta), epileptiform discharges, and/or an absent PDR. Absent PDR was used as a surrogate for encephalopathy as this marker is associated with worse 1‐year outcomes in pNMDARE [27]. Hospital length of stay (LOS) was defined as the duration, in days, a patient spent in the inpatient hospital, including the initial hospitalization at another facility prior to transfer to our service, if applicable, and before either discharge home or to the inpatient rehabilitation unit (IRU). LOS + IRU was defined as the duration, in days, a patient spent in both the hospital and the IRU.

### 2.3 Statistical Analysis

Chi‐squared statistic was employed for dichotomous variable comparisons, and Fisher's exact test was used for smaller group sizes (n<5). Mann–Whitney U test was employed to compare two groups of ordinal values or continuous numerical values with non‐parametric distributions. Kruskal–Wallis test was used to compare more than two groups of either ordinal values or non‐parametric continuous numerical values, and Two‐Way ANOVA was used to compare the interaction between more than two groups across time. Linear regression was used to compare the relationship between two continuous variables, and logistic regression for comparing binary classifications. Comparisons were considered statistically significant at *p*<0.05. Statistical analyses were performed with GraphPad Prism 10.4 (GraphPad Software, Boston, Massachusetts, USA).

### 2.4 Study Approval

The study was approved by the Institutional Review Board at Baylor College of Medicine and Texas Children's Hospital, Houston, TX (H‐35069).

## 3 Results

### 3.1 Demographics and Etiologies

**FIGURE 1:**
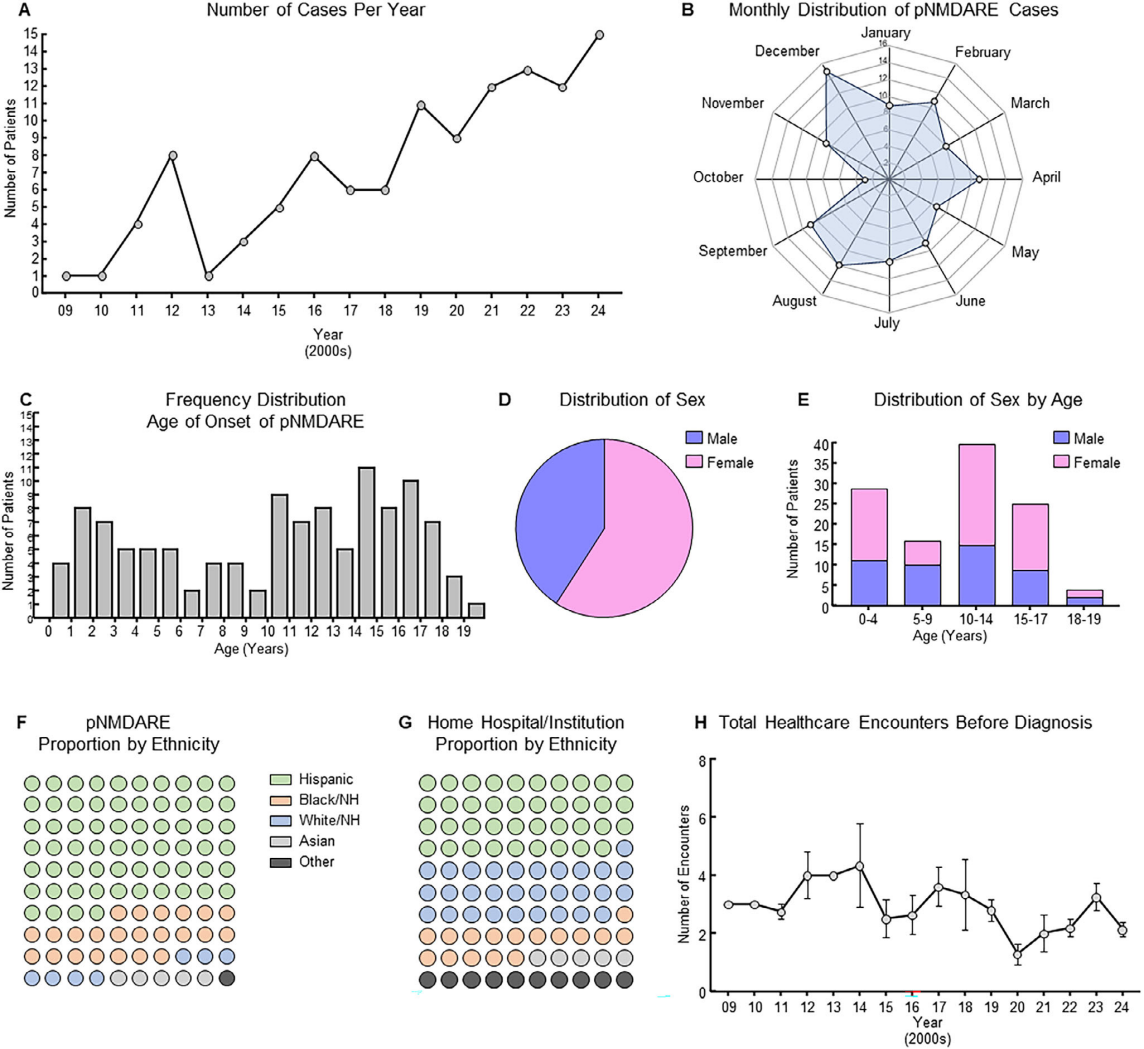
Basic demographics and change in frequency of pNMDARE over time. (A) Number of cases rises steadily from the first case in 2009 to 2024. (B) August and December are the most common months for a child to present with initial pNMDARE symptoms. (C) There is not a normal distribution of cases by childhood age. (D) Almost two‐thirds of all children are female, (E) but children <10 are roughly split 1:1 and >10 are predominantly female. (F) Two‐thirds of the children are Hispanic, (74/115, 64.3%) (G) which is disproportionately more than the home institution (39% Hispanic), city of Houston and Harris County (44%, data no shown). (H) The total number of healthcare encounters per patient prior to their pNMDARE diagnosis did not change from the initial patient in 2009 to 2024.

One hundred fifteen patients met the inclusion criteria for pNMDARE at Texas Children's Hospital (TCH) between 2009 and 2024. The annual number of diagnoses increased from one in 2009 to 15 in 2024 (Figure 1A). The seasonal distribution of cases presented a bimodal trend toward August/September and December (Figure 1B). Patients ranged from age 3.5 months to 19.8 years at the onset of their NMDARE symptoms, with an average age of 10.3 ± 0.5 years (Figure 1C). Almost two‐thirds (68/115; 59.1%) were female (Figure 1D); the proportion of female sex remained predominant across the span of ages (binned in increments of five years) except for ages 5–9 years, which was predominantly male sex (58.8%; Figure 1E). While the Houston Metropolitan Area (in which TCH resides) is comprised of 44% Hispanic people (36% non‐Hispanic, White and 17% Black) [28], and the TCH patient population roughly mirrors that of the Metropolitan Area (39%, unpublished data), a significantly greater proportion of pNMDARE patients were Hispanic (74/115, 64.4%, *p*<0.0001) and 26/115 (22.6%) were Black/non‐Hispanic (Figure 1F,G). Most patients (77/115; 67.0%) spoke English or were bilingual with English as their primary language, compared to 36/115 patients (31.3%) who spoke Spanish only and 2/115 patients (1.7%) who spoke a different language (Figure S1A). Because we observed our cohort was disproportionately represented by Hispanics, we aimed to identify potential confounders that may influence differences by ethnicity. ADI was obtained for each subject as a measure of socioeconomic disadvantage based on the neighborhood at the time of hospitalization with 1 being low disadvantage and 10 being high disadvantage. Two subjects did not have an ADI available to extract. 23/113 patients (20.3%) had an ADI of 1–3, 58/113 patients (51.3%) had an ADI of 4–7, and 32/113 patients (28.3%) had an ADI of 8–10 (Figure S1B). ADI was significantly lower (less socioeconomic disadvantage) in patients who self‐reported non‐Hispanic compared to Hispanic (2.6 ± 0.44 vs. 6.4 ± 0.29, *p*<0.01; Figure S1C). There was no difference in age by sex or race/ethnicity. The total number of healthcare encounters per patient prior to final diagnosis of pNMDARE remained relatively unchanged over time from an average of 3.0 ± 0.0 clinical encounters per patient prior to diagnosis in 2009–2010 to 2.1 ± 0.2 clinical encounters per patient prior to diagnosis in 2024 (Figure 1H).

**FIGURE 2:**
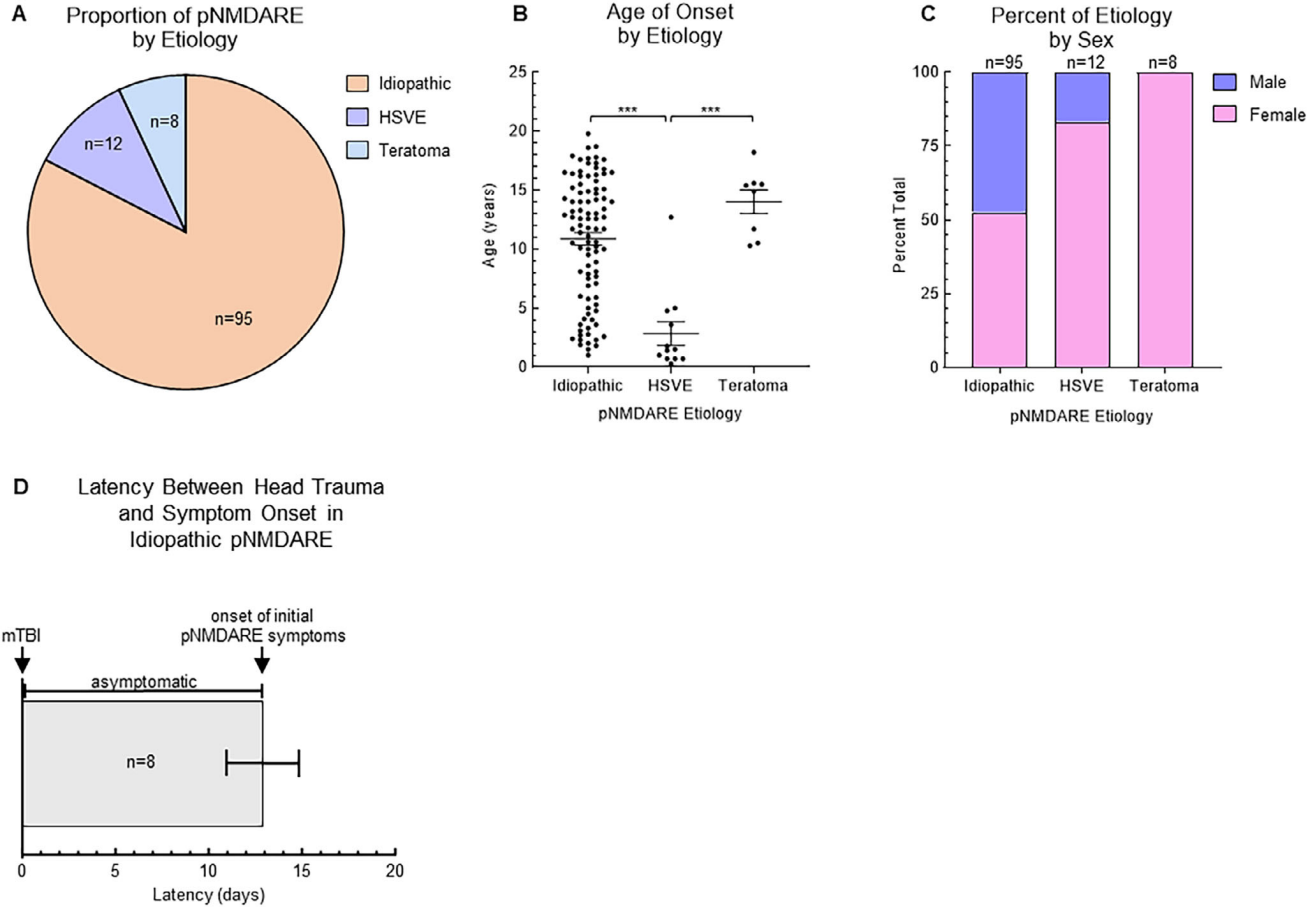
Characteristics of pNMDARE etiologies. (A) Most pNMDARE cases were idiopathic (82.6%), followed by recent HSVE (10.4%), and teratoma‐associated (7.0%). (B) Those with recent HSVE were younger (2.9 ± 1.0 years) than both idiopathic (10.9 ± 0.5 years) and teratoma‐associated cases (14.0 ± 1.0 years). (C) Most idiopathic cases were female (50/95, 52.6%), almost all HSVE cases were female (10/12, 83.3%), and all teratoma‐associated cases were female (8/8, which were all ovarian teratomas). (E) 8/115 (7.0%) patients presented 14.0 days (5–21 days) after mTBI/concussion. ***p<0.005 HSVE = herpes Simplex Virus Meningoencephalitis; mTBI = Mild Traumatic Brain Injury.

Twelve patients (10.4%) with pNMDARE were secondary to recent HSV‐1 or HSV‐2 ME (median 47.0 days, 16–98 days) prior to the onset of pNMDARE symptoms, which we previously published [29], eight (7.0%) were secondary to ovarian teratoma in females, and 95 (82.6%) were diagnosed with idiopathic pNMDARE (Figure 2A). The average age of each was 2.9 ± 1.0 years, 14.0 ± 1.0 years, and 10.9 ± 0.5 years respectively (Figure 2B). Female sex was predominant in 50/95 patients (52.6%) with idiopathic pNMDARE, 10/12 patients (83.3%) with HSVE‐associated pNMDARE, and 8/8 patients (100%) with teratoma‐associated pNMDARE (Figure 2C).

Notably, 8/115 patients (7.0%, all 8 from the idiopathic group) had a preceding mild traumatic brain injury (mTBI)/concussion prior to the onset of their pNMDARE symptoms. One patient exhibited brief loss of consciousness and 4/8 sought medical care on the day of the mTBI, all obtaining a normal head CT and 2/4 requiring sutures to their head/facial lacerations. The mTBIs were 14.0 days (5–21 days) prior to the onset of pNMDARE symptoms (such as focal weakness/sensory disturbance, chorea, seizures, behavioral abnormalities or psychosis). Strikingly, parents or caregivers reported *no* pNMDARE symptoms prior to the head trauma in any of these children and observed completely typical behavior after the head trauma up until the onset of symptoms, as reported roughly two weeks later (Figure 2D).

### 3.2 Application of Exclusion Criteria for the Remainder of the Study

**FIGURE 3:**
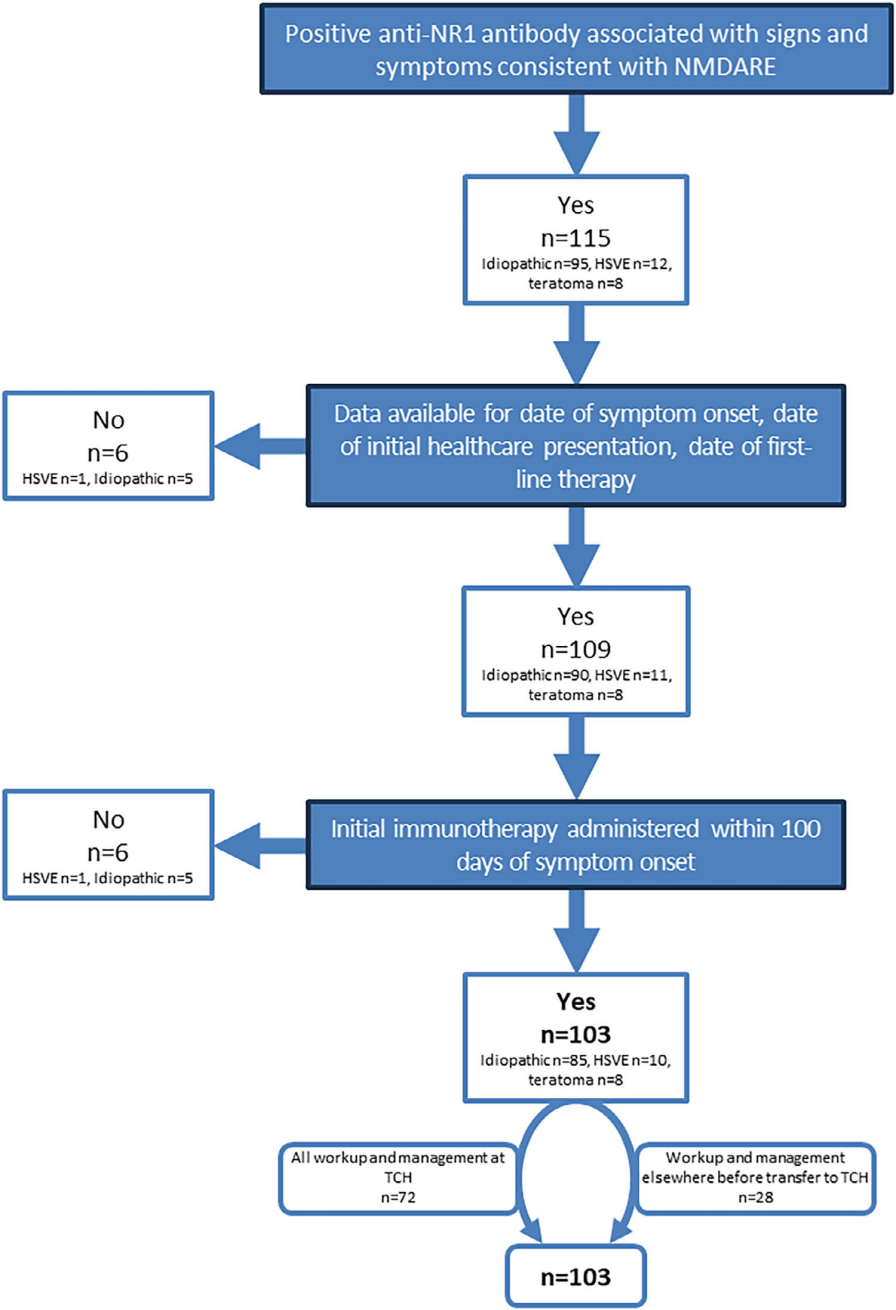
Flowchart applying the exclusion criteria.

The remainder of the study focuses on presenting symptoms, workup including laboratory evaluation, imaging, and EEG, treatment, and outcomes. Not all subjects had all pertinent parameters available for data extraction. Further, some subjects had extremely prolonged medical courses with significant delays in diagnosis and treatment (up to nearly two years in some cases) which confounded the biological markers we sought to characterize. We therefore applied exclusion criteria to the cohort in order to select for subjects with the most complete datasets available (n = 109) and those that were diagnosed and treated with first‐line immunotherapy within 100 days (n = 103; Figure 3). Among the 12 excluded subjects with known dates of symptom onset and interventions, the average latency to first‐line therapy was 524.9 ± 244.7 days (n = 8). Most patients (74/103; 71.8%) received their initial workup and management at our institution. The remaining patients received their initial workup and management for pNMDARE elsewhere and were transferred to us for the next steps in care after having received a diagnosis and immunotherapy. There was, therefore, some variation in practice patterns. There was no difference in demographic variables described above (ethnicity, age, sex) between those that had their complete workup and management at our hospital vs. those that transferred to us after their workup and initial management. The remainder of the study focuses on this cohort of 103 subjects.

### 3.3 Presenting Symptoms

**FIGURE 4:**
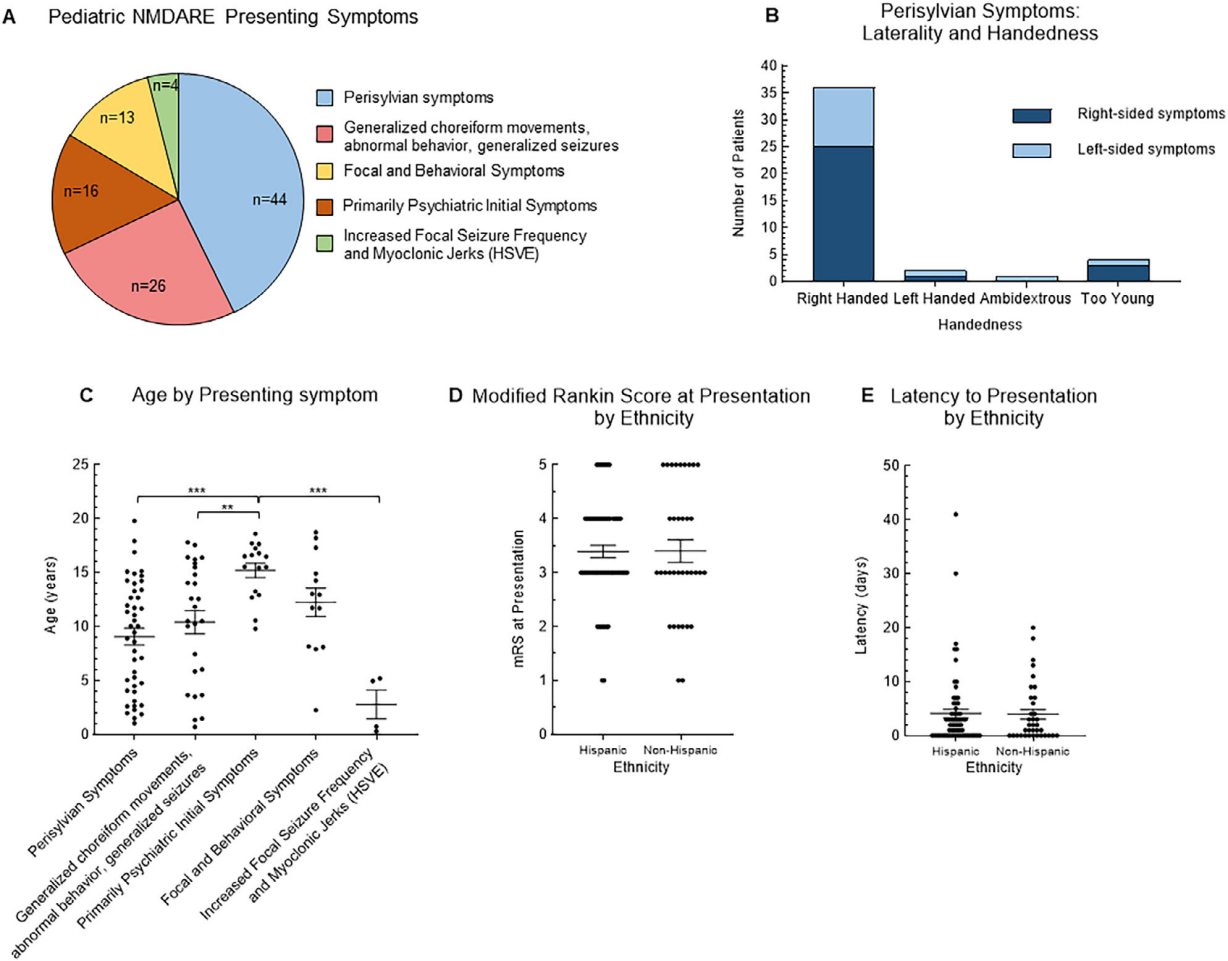
Characteristics of pNMDARE presentation. (A) 44 (42.7%) presented with a specific stroke‐like focal cortical phenotype that localized to the perisylvian region as their initial symptomology prior to other pNMDARE symptoms. 26 (25.2%) presented with generalized choreiform movements, abnormal behavior, or generalized seizures, 16 (15.5%) presented with focal and behavioral symptoms, 13 (12.6%) presented with primarily psychiatric initial symptoms, 4 (3.9%) presented with increased focal seizure frequency and myoclonic jerks in the setting of recent HSVE. (B) Of the 44 children who presented with perisylvian symptoms, 5 were too young for known hand dominance. 36/39 were right hand dominant (92.3%); 25/36 (69.4%) of them had symptoms on the ipsilateral side as their hand dominance. (C) Children with primarily psychiatric initial symptoms were significantly older (15.2 ± 0.6 years) than those who presented with perisylvian symptoms (9.1 ± 0.8 years, *p*<0.005), generalized choreiform/generalized seizures (10.4 ± 1.1 years, *p*<0.005), and increased focal seizure frequency with myoclonic jerks (2.8 ± 1.3 years, *p*<0.005). There was no difference in (D) presenting mRS or (E) latency to presentation by ethnicity. **p<0.01, ***p<0.005. HSVE = herpes Simplex Virus Meningoencephalitis; NH = non‐Hispanic; mRS = Modified Rankin Scale.

Forty‐four patients (42.7%) presented with a specific stroke‐like focal cortical phenotype that localized to the perisylvian region as their initial symptomology prior to the onset of other classic pNMDARE features such as seizures, psychosis, and movement disorder (Figure 4A). These initial symptoms are localizable neurological findings, such as isolated right‐sided upper and lower extremity weakness with sensory disturbance described best as dysesthesia associated specifically with expressive aphasia. It was unclear which side of the body was affected in one patient. Right‐sided somatic symptoms were more common than left‐sided somatic symptoms (67.4% vs. 32.6%, respectively). Nearly two‐thirds (26/43; 60.5%) of patients with perisylvian symptoms were affected on the ipsilateral side to their hand‐dominance with 12/43 patients (27.9%) exhibiting the symptoms contralateral to their hand‐dominance and 4 possessing unknown hand dominance due to young age (Figure 4B). An additional 13/103 children (12.6%) initially exhibited a confluence of focal symptoms (i.e., unilateral dysesthesias, weakness) *and* psychiatric or behavioral symptoms simultaneously, prior to the onset of other symptoms such as seizures. Approximately one‐quarter (26/103; 25.2%) of patients presented with generalized symptoms such as bilateral choreiform movements, generalized seizures, and/or abnormal behavior (but not psychosis) as their initial symptomology while 16/103 patients (15.5%) presented with isolated psychosis (delusions, auditory or visual hallucinations) prior to the onset of other movement disorders or seizures. The median latency between the onset of isolated psychosis to other conventional pNMDARE symptoms was 3.5 days (range 0–10 days, 25^th^‐75^th^ percentile 0.75–6.5 days, and the latency was not known in 4 patients). The average age of those with isolated psychiatric symptoms (15.2 ± 0.6 years) was significantly older than those with perisylvian symptoms (9.1 ± 0.8 years, *p*<0.005), generalized choreiform/generalized seizures (10.4 ± 1.1 years, *p*<0.005), and increased focal seizure frequency with myoclonic jerks (2.8 ± 1.3 years, *p*<0.005; Figure 4C). Four patients (3.9%) presented with increased focal seizure frequency and the emergence of myoclonic jerks, all in the setting of recent HSVE. Two patients (1.9%) had unknown initial symptoms. The proportion of those who self‐identified as Hispanic was similar among each of the presenting symptom groups (about two‐thirds, p>0.05 for each comparison).

Because we observed differences in pNMDARE biomarkers based on self‐identified race and ethnicity, we sought to identify potential drivers for the differences such as latency to presentation and socioeconomic determinants of health. Modified Rankin Scale (mRS) was extracted from the clinical data at presentation. We compared the clinical severity and latency at which different demographic groups presented to healthcare for the first time. There was an observed trend that White/non‐Hispanic patients presented to healthcare professionals with a lower presenting mRS compared to Hispanic and Black/non‐Hispanic (2.5 ± 0.5 vs 3.4 ±0.1 and 3.5 ± 0.2, respectively, data not shown) and that latency from onset of symptoms to healthcare presentation trended shorter in White/non‐Hispanic patients compared to Hispanic, Black/non‐Hispanic, and Asian patients; data not shown), but are underpowered to detect true differences. There was no difference in presenting mRS latency to presentation by ethnicity (Figure 4D,E). There were also no differences in mRS at presentation or latency to presentation between ages, sexes, presenting symptom groups, language spoken (Figure S1D), or by ADI (Figure S1E).

### 3.4 Workup

#### 3.4.1 Lumbar Puncture and CSF Analysis

**FIGURE 5:**
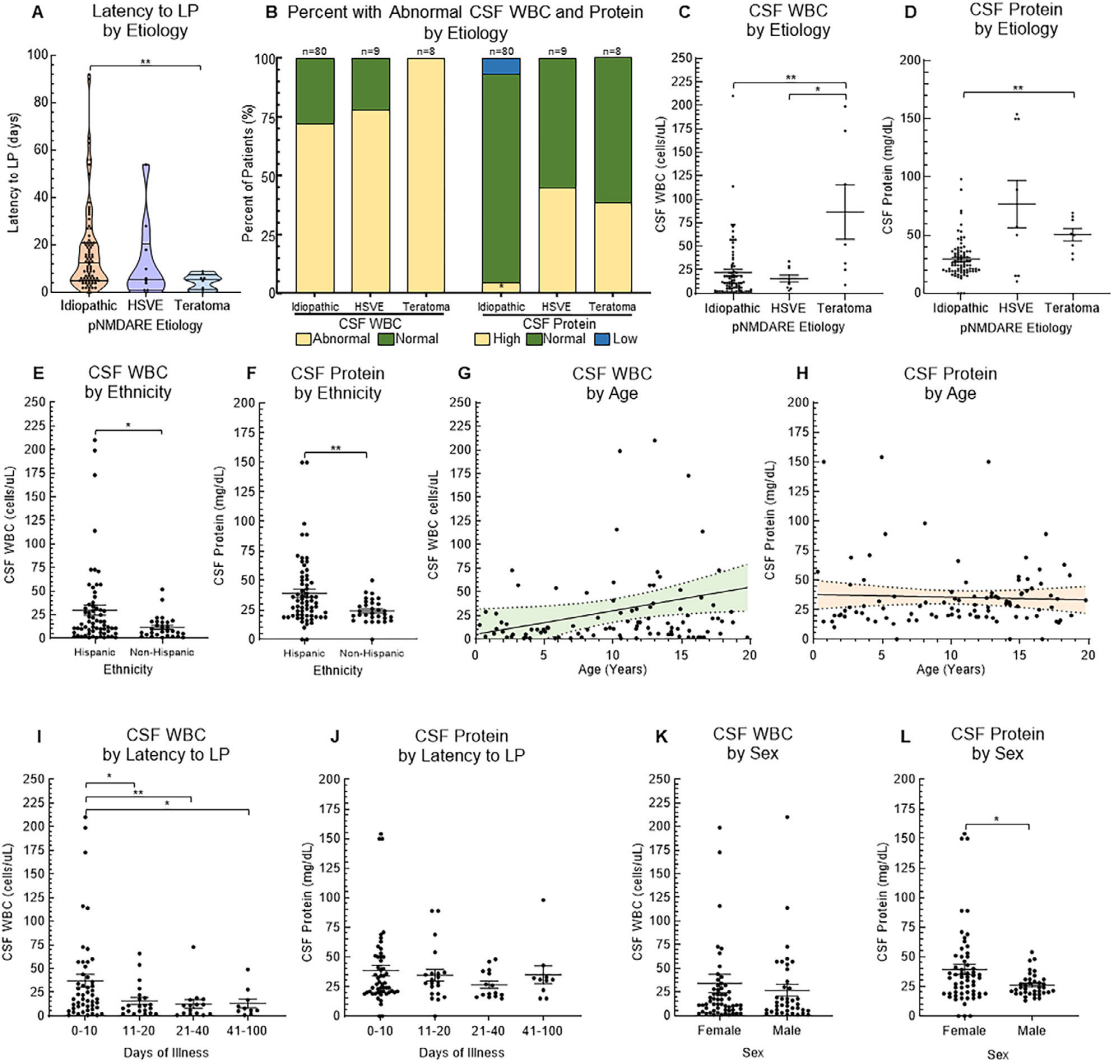
Lumbar puncture and CSF characteristics. (A) Patients with teratoma had an initial LP earlier (4.6 ± 1.2 days) than idiopathic cases (12.7 ± 5.4 days). (B) All teratoma‐associated cases had abnormal CSF WBC while most cases of idiopathic and HSVE had abnormal CSF WBC (57/80, 71.3%, and 7/9, 77.8%, respectively). Almost all idiopathic patients had normal CSF protein (71/80, 88.8%), and about half of HSVE and teratoma‐related patients had normal CSF protein (5/9, 55.6%, and 5/8, 62.5%, respectively). The idiopathic group had a smaller proportion of high CSF protein than the other two groups (*p*<0.05). (C) CSF WBC was higher in those with teratoma (86.6 ± 28.9) than both idiopathic (22.1 ± 3.4) and HSVE (15.7 ± 3.8). (D) Idiopathic patients had lower CSF protein (29.2 ± 1.9 mg/dL) and teratoma‐associated pNMDARE (50.4 ± 5.3 mg/dL). (E) Hispanic patients had higher CSF WBC (30.1 ± 5.4 cells/uL) than non‐Hispanic patients (11.8 ± 2.2 cells/uL), and (F) the CSF protein was higher in the Hispanic group (38.8 ± 3.6 mg/dL) than the non‐Hispanic group (24.4 ± 1.8 mg/dL, *p*<0.005). (G) There was a positive linear correlation between age and CSF WBC (F = 4.7, p<0.05, r2 = 0.05, *n* = 97), while (H) there was no relationship between age and CSF protein. (I) CSF WBC was higher in those with an initial LP obtained between 0 and 10 days after symptom onset (37.1 ± 6.8 cells/uL) than 11–20 days (15.9 ± 3.8 cells/uL), 21–40 days (12.7 ± 4.6 cells/uL), and 41–100 days (13.2 ± 4.7 cells/uL), while (J) CSF protein was not different based on latency to LP. (K) CSF WBC was not different between females and males, while (L) CSF protein was higher in females (39.6 ± 4.3 mg/dL) than males (26.1 ± 1.6 mg/dL). *p<0.05, **p<0.01, ***p<0.005. LP = Lumbar Puncture; HSVE = herpes Simplex Virus Meningoencephalitis.

Almost all patients (101/103) had an LP as part of their workup; 97/101 had *initial* AE workup CSF variables available for extraction (some transferred patients did not have initial CSF data available). The median latency from AE symptom onset to LP was significantly shorter in teratoma‐associated pNMDARE compared to idiopathic (Figure 5A). Latency to LP did not differ between sexes or race/ethnicities. Most patients (72/97, 74.2%) had abnormal CSF WBC (> 5 cells/µL). The proportion of patients with abnormal CSF WBC did not differ between idiopathic, HSVE, and teratoma‐associated pNMDARE (57/80, 7/9, and 8/8, respectively, Figure 5B). In contrast, CSF protein was low (<15 mg/dL) in 6/97 patients (6.2%, all idiopathic patients), normal (15–60 mg/dL) in 80/97 patients (82.5%), and high (>60 mg/dL) in 11/97 patients (11.3%). A lower proportion of idiopathic pNMDARE subjects had high CSF protein (Figure 5B, *p*<0.05). However, the *average* CSF WBC was significantly higher in those with teratoma‐associated pNMDARE than those with idiopathic or HSVE‐associated pNMDARE (86.6 ± 28.9 vs. 22.1 ± 3.4, *p*<0.01, and 15.7 ± 3.8, *p*<0.05, respectively; Figure 5C), and the CSF protein was significantly lower in those with idiopathic compared to teratoma‐associated pNMDARE (29.2 ± 1.9 vs. 50.4 ± 5.3 mg/dL, respectively, *p*<0.01, Figure 5D). Hispanic patients had a greater CSF WBC than non‐Hispanic patients (30.1 ± 5.4 cells/uL vs. 11.8 ± 2.2 cells/uL, *p*<0.01; Figure 5E) and CSF protein (38.8 ± 3.6 mg/dL vs. 24.4 ± 1.8 mg/dL, *p*<0.01; Figure 5F). Due to the observed difference in CSF parameters by self‐reported ethnicity, we evaluated potential drivers of this outcome by socioeconomic disadvantage. Neither CSF WBC (Figure S1F) nor CSF Protein were different by grouped ADI, nor was there a difference by transfer status (Figure S1G). There was a positive linear correlation between age and CSF WBC (F = 4.7, *p*<0.05; Figure 5G) while CSF protein was not different across ages (*F* = 0.20, *p* = 0.65; Figure 5H).

We initially performed a linear regression correlating CSF WBC and protein to latency to LP, which had no relationship. However, when categorizing, CSF WBC was highest between 0 and 10 days from symptom onset compared to 11–20, 21–40, and 41–100 days from symptom onset (37.1 ± 6.8 cells/uL vs. 15.9 ± 3.8 cells/uL, 12.7 ± 4.6 cells/uL and 13.2 ± 4.7 cells/uL, respectively, *p*<0.05; Figure 5I) while CSF protein did not differ by latency to LP (Figure 5J). There was no difference in CSF WBC count between sexes (Figure 5K), but average CSF protein was significantly higher in females than males (39.6 ± 4.3 mg/dL vs. 26.1 ± 1.6 mg/dL; *p*<0.05; Figure 5L). There was no difference in CSF WBC when comparing those that were worked up and managed at our institution (21.2 ± 2.9 cells/uL) vs. those who were managed elsewhere prior to transfer to our institution (24.1 ± 6.5 cells/uL, p = 0.65).

### 3.5 Antibody Analysis

**FIGURE 6:**
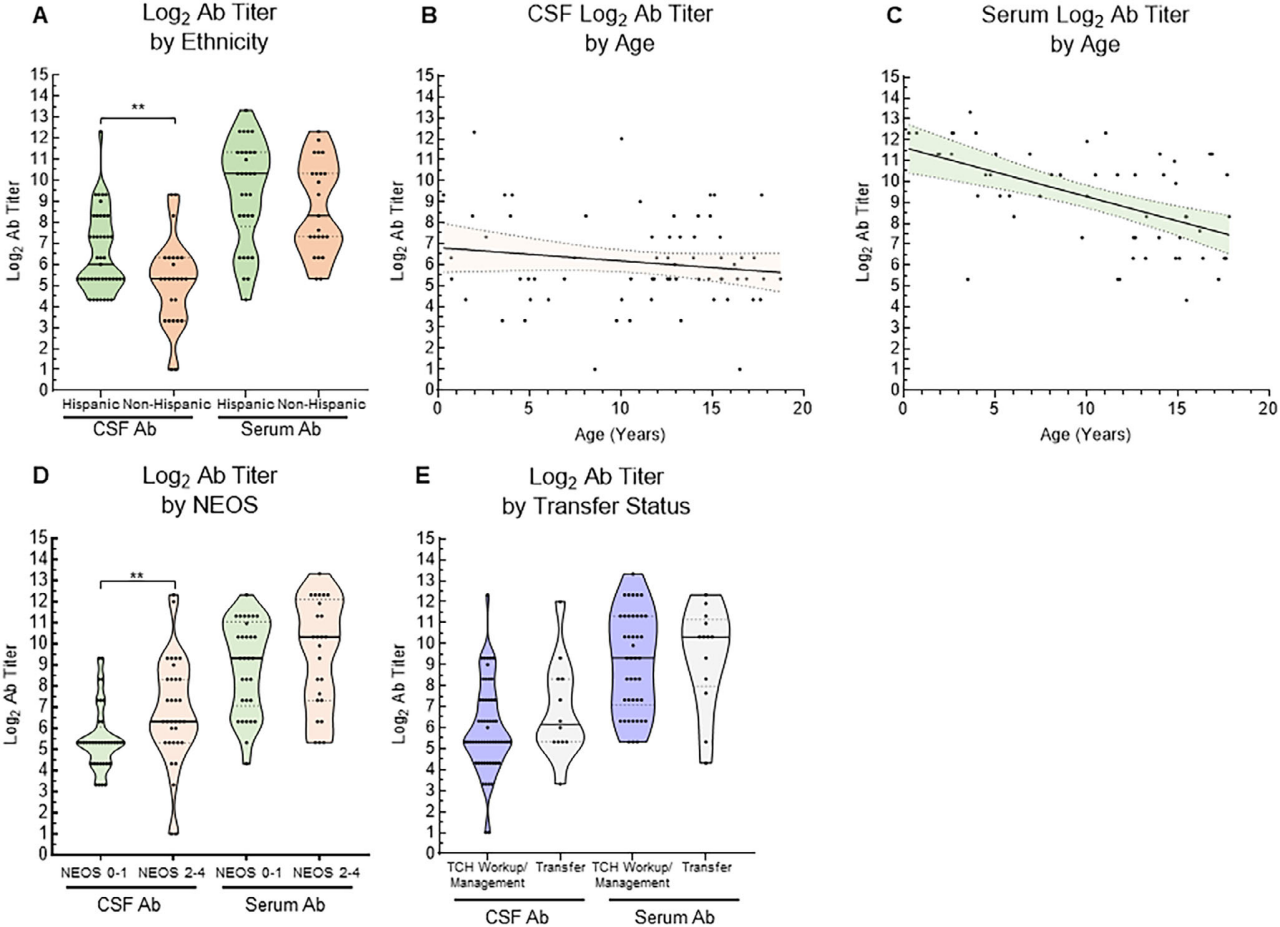
Log_2_‐transformed anti‐NMDAR antibody titers. (A) Hispanic patients had higher CSF Ab titers (6.5 ± 0.3) than non‐Hispanic patients (5.2 ± 0.4), while serum Ab titers were not different between ethnicities. (B) There was no relationship between age and CSF Ab titer, but (C) there was a negative linear correlation between age and serum Ab titer (*F* = 21.3, *p*<0.0001, *r*2 = 0.29). (D) CSF Ab titer was higher in those with NEOS 2–4 (6.9 ± 0.4) than NEOS 0–1 (5.3 ± 0.3), however there was no difference in serum Ab titer. (E) There was no difference in CSF or serum Ab titer based on if subjects were worked up and managed at TCH vs worked up elsewhere before transfer to TCH. *p<0.05, **p<0.01, ***p<0.005. NEOS = NMDARE 1‐Year Functional Status.

Seventy initial CSF Ab titers and 55 initial serum Ab titers were available for further analyses (while 31 CSF Ab titers resulted as “positive” without a titer). Hispanic patients had significantly higher Log_2_ CSF Ab titers compared to non‐Hispanic patients (6.5 ± 0.3 vs. 5.2 ± 0.4, respectively; *p*<0.01), while serum Ab titers were not different between ethnicities (Figure 6A), although there was no difference in either Log_2_ CSF or serum Ab titers when compared by ADI group (Figure S1H) or by transfer status (Figure S1I). There was also no difference in Log_2_ Ab titers in either CSF or serum when comparing sex, etiology, or latency to NMDAR Ab acquisition. There was no correlation between age and CSF Log_2_ Ab titers (Figure 6B), but there was a negative correlation between age and serum Log_2_ Ab titers (*F* = 21.3, *p*<0.0001; Figure 6C). Patients with NEOS 0–1 had significantly lower CSF Log_2_ Ab titers compared to those with NEOS 2–4 (5.3 ± 0.3 vs. 6.9 ± 0.4, *p*<0.01), while there was no difference in serum Ab titers (Figure 6D). There was no difference in CSF or serum Log_2_ Ab titers between those that had their complete workup and management at our institution vs. those who were transferred to our institution after workup and initial management (Figure 6E).

### 3.6 Imaging Modalities and Electroencephalogram

All 10 patients with prior HSVE had an abnormal MRI brain and EEG at the time of their AE workup. Of the remaining 93 non‐HSVE patients, one patient had a contraindication to obtaining an MRI brain. Most (66/92, 71.7%) non‐HSVE MRIs of the brain were unremarkable at the onset of AE workup (Table 1). There was no difference in latency from symptom onset to the initial MRI obtained between the abnormal and unremarkable MRIs. Excluding HSVE, there was no difference in the proportion of patients with unremarkable initial MRI between sexes, race/ethnicities, ages, etiologies, or presenting symptoms. There was no difference in mRS at presentation or Log_2_ CSF and serum Ab titers between patients with initially abnormal vs. unremarkable MRI.

**TABLE 1:** Initial MRI Characteristics by Demographics, Etiologies, Presenting Symptoms, and Titers.

	Abnormal MRI	Unremarkable MRI	p value	Binary Logistic Regression
**Total Initial MRIs** (excluding HSVE) *n* = 92	26	66		
**Latency to MRI (days)**	8 days, 1–91 days	8 days, 0–63 days	0.82	
**Sex, n** Female Male	12 14	38 28	0.32	
Age				ROC AUC 0.55, p = 0.42
**Ethnicity, n** Hispanic non‐Hispanic	15 11	45 21	0.34	
**Etiology, n** Idiopathic Teratoma HSVE	22 4 10	62 4 0	0.22 (idiopathic v teratoma)	
**Presenting Symptom, n** Perisylvian symptoms Generalized choreiform Isolated Psychiatric Focal Behavioral	11 7 6 2	32 14 9 11	0.50	
**Presenting mRS** ^A^	3.3 ± 0.2	3.3 ± 0.1	0.83	
**CSF Log_2_ Ab Titer** ^B^	6.0 ± 0.6 *n* = 19	5.8 ± 0.3 *n* = 45	0.65	
**Serum Log_2_ Ab Titer** ^C^	6.9 ± 1.1 *n* = 17	6.9 ± 0.6 *n* = 49	0.99	

There were 92 patients with non‐HSVE‐related NMDARE that had an MRI of the brain with a known result (unremarkable vs abnormal). All latencies are expressed as median, range in days, and all other continuous numerical values are expressed as the mean ± standard error of the mean. HSVE is excluded from initial analysis due to all HSVE‐associated cases having abnormal MRI.

^A^ Excludes unknown presenting mRS (n = 2).

^B^ Excludes unknown CSF Ab titer.

^C^ Excludes unknown serum Ab titer.

About half (48/92, 52.2%) of non‐HSVE patients with data available for analyses had an abnormal initial EEG (Table 2); the latency from symptom onset to initial EEG was not different between groups. There was no difference in the proportion of abnormal EEG between sexes, ethnicities, ages, etiologies, MRI results, or presenting symptoms. Those with an abnormal initial EEG had a higher mRS at presentation compared to those with an unremarkable EEG (3.5 ± 0.1 vs. 3.0 ± 0.1, *p*<0.05). Log_2_ Ab titers in CSF or serum was not different between patients by EEG results. Among those with an initially unremarkable EEG (n = 44, median latency to initial EEG 7.0 days, 0–47 days), 22 eventually converted to an abnormal EEG 11.0 days, 3–45 days after symptom onset, while 19 patients never had an abnormal EEG (despite 1.9 ± 0.3 repeat EEGs per patient, range 1–5). Overall, excluding those with HSVE, most patients (72/92, 78.3%) *eventually* had an abnormal EEG with a median latency of 9.0 days (1–45 days) and required 1.9 ± 0.3 EEG attempts until an abnormal EEG was obtained.

**TABLE 2:** Initial EEG Characteristics by Demographics, Etiologies, Presenting Symptoms, and Titers.

	Abnormal EEG	Unremarkable EEG	p value	Binary Logistic Regression
**Total Initial EEG** (excluding HSVE) n = 92	48	44		
**Latency to EEG (days)**	8.5 days, 1–74 days	7.0 days, 0–47 days	0.33	
**Sex, n** Female Male	24 24	27 17	0.27	
Age				ROC AUC 0.58 p = 0.18
**Ethnicity, n** Hispanic non‐Hispanic	30 18	29 15	0.73	
**Etiology, n** Idiopathic Teratoma HSVE	45 3 10	39 5 0	0.47 (idiopathic v teratoma)	
**Presenting Symptom, n** Perisylvian symptoms Generalized choreiform Isolated Psychiatric Focal Behavioral	25 10 5 8	18 11 10 5	0.34	
**Presenting mRS** ^A^	3.5 ± 0.1	3.0 ± 0.1	0.03	
**CSF Log_2_ Ab Titer** ^B^	5.9 ± 0.4 *n* = 35	6.0 ± 0.4 *n* = 29	0.98	
**Serum Log_2_ Ab Titer** ^C^	8.9 ± 0.4 *n* = 31	9.1 ± 0.5 *n* = 21	0.71	

There were 92 patients non‐HSVE‐related‐NMDARE who had a known result of their initial EEG of the brain (unremarkable vs abnormal). All latencies are expressed as median, range in days, and all other continuous numerical values are expressed as the mean ± standard error of the mean. HSVE is excluded from initial analysis due to all HSVE‐associated cases having abnormal EEG.

^A^ Excludes unknown presenting mRS (n = 2).

^B^ Excludes unknown CSF Ab titer.

^C^ Excludes unknown serum Ab titer.

### 3.7 Treatment

**FIGURE 7:**
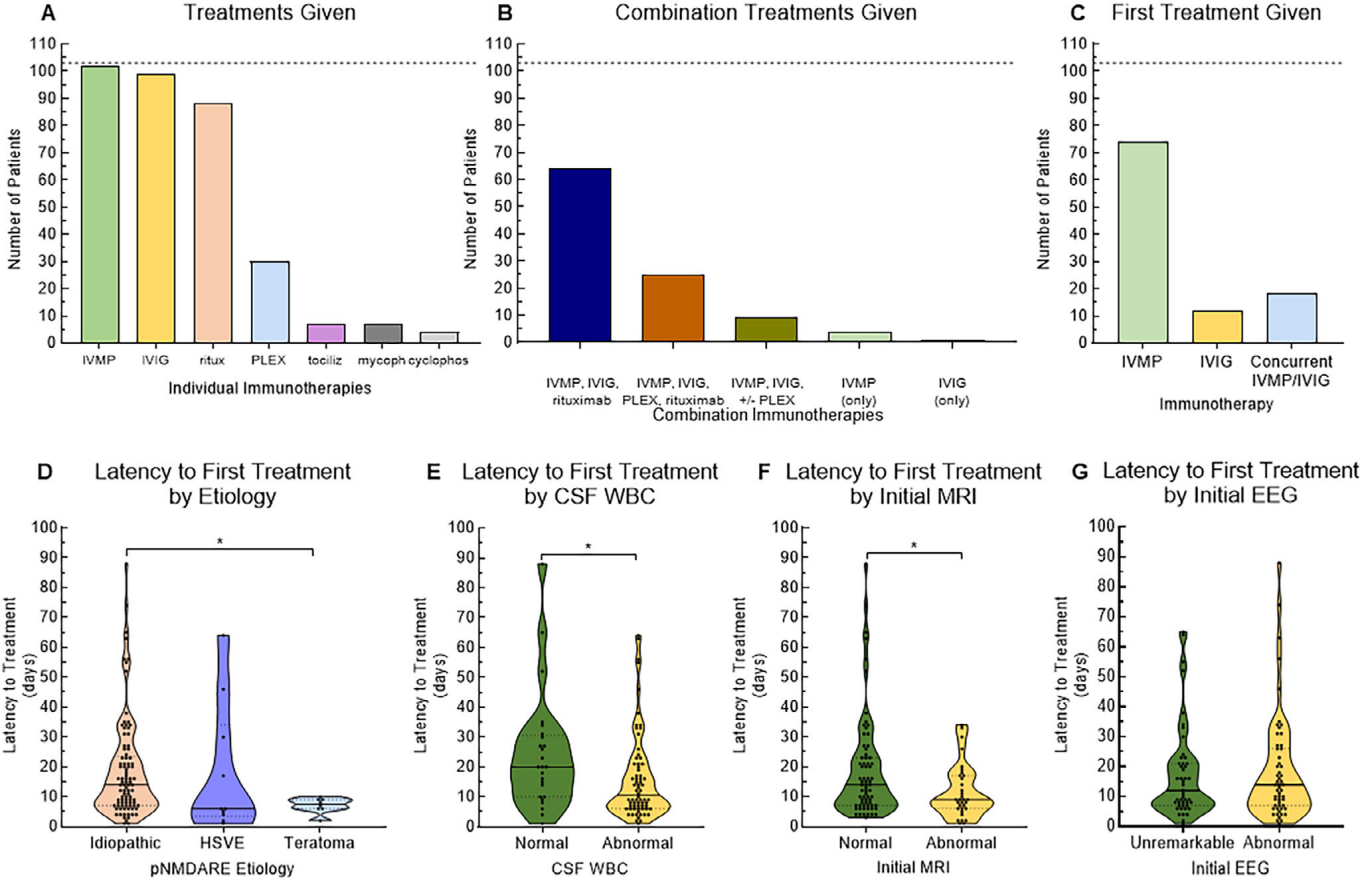
Treatments given for pNMDARE and their timeline. (A) Of 103 patients, 102 received IVMP, 99 received IVIG, 88 received rituximab, 30 had PLEX performed, 7 received tocilizumab, 7 received mycophenolate, and 4 received cyclophosphamide. (B) 64 patients received the combination of IVMP, IVIG, and rituximab without PLEX, and 25 received IVMP, IVIG, and rituximab with PLEX. (C) IVMP was the first treatment given in 74 patients. (D) Those with teratoma had an initial treatment earlier (7.5 days, 2–10 days) than those with idiopathic pNMDARE (19.4 days, 1–88 days). (E) Those with abnormal CSF WBC received initial treatment earlier (16.0 days, 1–64 days) than patients with normal CSF WBC (24.4 days, 1–88 days), (F) as did those with abnormal MRI brain (11.7 days, 1–34 days) compared to normal MRI brain (20.0 days, 3–88 days), while (G) EEG results were not associated with latency to initial treatment. *p<0.05, **p<0.01, ***p<0.005. IVMP = intravenous methylprednisolone; IVIG = intravenous immunoglobulin; ritux = rituximab; PLEX = plasma exchange; tociliz = tocilizumab; mycoph = mycophenolate; cyclophos = cyclophosphamide; HSVE = herpes simplex virus meningoencephalitis.

Almost all patients received high‐dose intravenous methylprednisolone (IVMP; 102/103, 99.0%), intravenous immunoglobulin (IVIG; 99/103, 96.1%), and rituximab (88/103, 85.4%), while 30/103 (29.1%) received plasmapheresis/plasma exchange (PLEX, based on ICU course and severe dysautonomia, per hospital protocol), 7/103 (6.8%) received tocilizumab, 7/103 (6.8%) received mycophenolate, and 4/103 (3.9%) received cyclophosphamide (Figure 7A). All seven patients who received tocilizumab had already completed IVMP + IVIG + rituximab and were considered medically refractory as the rationale for initiating tocilizumab. All eight patients with an identified teratoma had the tumor resected in addition to IVMP + IVIG + rituximab. The most common combinations of immunotherapies were IVMP + IVIG + rituximab ± an additional non‐PLEX therapy (64/103, 62.1%), IVMP + IVIG + rituximab + PLEX ± an additional therapy (25/103, 24.2%), and IVMP + IVIG ± PLEX or mycophenolate (9/103, 8.7%, Figure 7B). Seventy‐six patients received IVMP first (73.8%), 12 received IVIG first (11.7%), and 15 received IVMP + IVIG concurrently starting the same day (14.6%, Figure 7C).

The latency from symptom onset to first treatment was significantly less in those with teratoma‐associated pNMDARE compared to the idiopathic group (7.5 days (2–10 days) vs. 15.0 days (1–88 days), *p*<0.05; Figure 7D). We explored potential explanations for patients with teratoma‐associated pNMDARE to receive IVMP earlier than other patients; the latency to presentation and the initial clinical severity of disease (assessed by mRS at presentation) were not different between the three etiological groups. However, there was a significantly increased latency from symptom onset to first treatment in those with normal CSF WBC (0–5 cells/uL) compared to abnormal CSF WBC (20.0 days (1–88 days) vs. 10.5 days (1–64 days), *p*<0.01; Figure 7E). Those with abnormal initial MRI had lower latency to first treatment (9.0 days, 1–34 days) compared to those with normal initial MRI (15.0 days, 3–88 days, *p*<0.05; Figure 7F), while there was no difference in latency to treatment based on initial EEG result (Figure 7G).

### 3.8 Outcomes

**FIGURE 8:**
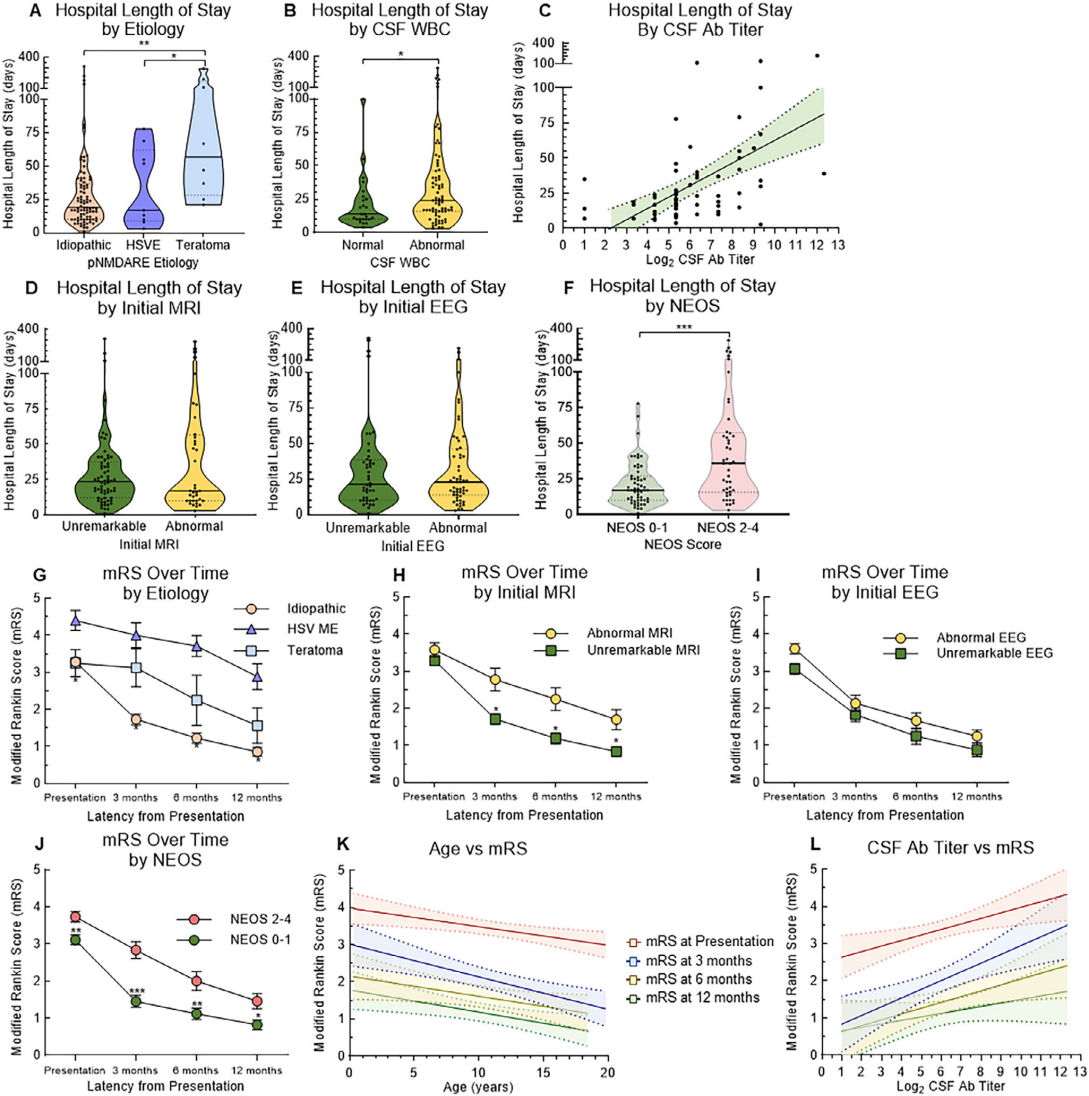
Clinical severity and outcomes. (A) Hospital length of stay was not different between those who presented within 0–7 days vs. 8+ days. (B) Those with teratomas had longer hospital LOS (median 57.0 days, 21–289 days) than both idiopathic (median 19.0 days, 1–313 days) and HSVE (17.0 days, 3–78 days), and (C) those with abnormal CSF WBC had longer hospital LOS (median 24.0 days, 3–289 days) than those with normal CSF WBC (median 14.0 days, 4–100 days). (D) There was a positive linear correlation between CSF Ab titer and hospital LOS (*F* = 26.1, *p*<0.0001, r^2^ = 0.28). Neither (E) initial MRI result nor (F) initial EEG results were associated with hospital LOS. (G) Patients with NEOS 0–1 had significantly shorter hospital LOS (median 17.0 days, 1–78 days) than those with NEOS 2–4 (median 36.0 days, 3–289 days). (H) mRS at presentation was lower in those with idiopathic pNMDARE compared to HSVE‐associated pNMDARE at presentation, 3, 6, and 12 months after the onset. (I) mRS at presentation was the same between those with abnormal initial MRI and unremarkable initial MRI, but was lower in those with unremarkable initial MRI at 3, 6, and 12 months, while (J) those with an unremarkable initial EEG had a significantly lower mRS at presentation (3.1 ± 0.1 vs. 3.6 ± 0.1, *p*<0.01)—but no difference at follow up times points—by initial EEG result. (K) Those with NEOS score 0–1 had lower mRS at presentation, 3, 6, and 12 months compared to those with NEOS score 2–4. (L) There was a negative linear correlation between age and mRS at presentation (*F* = 14.4, *p* < 0.001, *R* ^2^ = 0.13, *n* = 101), 3 months (*F* = 13.2, *p* < 0.001, *R* ^2^ = 0.12, *n* = 96), 6 months (*F* = 6.7, *p* < 0.05, *R* ^2^ = 0.08, *n* = 81), and 12 months (*F* = 8.7, *p* < 0.01, *R* ^2^ = 0.10, *n* = 80). (M) There is a positive linear correlation between Log_2_ CSF Ab titer and mRS at presentation (*F* = 8.1, *p* < 0.01, *R* ^2^ = 0.11, *n* = 69), 3 months (*F* = 12.1, *p* < 0.001, *R* ^2^ = 0.16, *n* = 66), and 6 months (F = 5.5, p < 0.05, *R* ^2^ = 0.10, *n* = 55). *p<0.05, **p<0.01, ***p<0.005.

Median LOS + IRU for the 103 subjects 22.0 days (1–313 days); 45/103 patients (43.7%) spent time in the TCH IRU for a median of 21.0 days (7–86 days). There were no differences in LOS based on sex, age, race, ethnicity, or presenting symptoms. Patients with teratoma‐associated pNMDARE had a significantly longer Hospital LOS compared to either the idiopathic group or HSVE groups (57.0 days, 21–289 days vs. 19.0 days, 1–313 days and 17.0 days, 3–78 days, *p*<0.05; Figure 8A). Patients with normal CSF WBC had significantly shorter LOS compared to those with abnormal CSF WBC (14.0 days, 4–100 days vs. 24.0, 3–289 days, *p*<0.05; Figure 8B). There was a positive linear correlation between CSF Log_2_ Ab titer and LOS (F = 26.1, *p*<0.0001, r^2^ = 0.28; Figure 8C), while there was no correlation between serum Ab titer and LOS. There was no difference in LOS by initial MRI or initial EEG (Figure 8D,E), but those with NEOS 0–1 had significantly shorter LOS compared to NEOS 2–4 (17.0 days, 1–78 days) vs. 36.0 days (3–289 days), *p*<0.001; Figure 8F).

There was a loss of 12‐month outpatient clinic follow‐up in patients who generally did clinically well. There were 102 mRS scores at presentation and 80 mRS scores at 12 months. For example, many patients who had a presumed mRS of zero at 6 or 12 months did not continue follow up with our outpatient group, thus the 12‐month mRS is likely overestimated by inclusion of only subjects who continued to follow up in clinic. Nevertheless, we report mRS at presentation, 3, 6, and 12 months among those who did follow up. mRS at presentation was significantly lower in both idiopathic and teratoma NMDARE groups compared to HSVE. Idiopathic remained significantly lower over the following 12 months (0.9 ± 0.1 vs. 2.6 ± 0.4, *p*<0.05; Figure 8G). Presenting mRS was the same among those with abnormal MRI and unremarkable MRI, but those with unremarkable MRI had lower mRS through 12 months (0.8 ± 0.1 vs. 1.6 ± 0.3, *p*<0.05; Figure 8H), while mRS was not different based on initial EEG result at any time point (Figure 8I). Presenting mRS was lower in those with NEOS 0–1 compared to NEOS 2–4, and it was significantly lower at 3 months (1.3 ± 0.1 vs. 2.6 ± 0.2, *p*<0.01) and at 6 months (1.0 ± 0.1 vs. 1.7 ± 0.2, *p*<0.05) then the mRS gap narrowed through 12 months (Figure 8J).

We next correlated age and CSF Ab titer to mRS at each timepoint with linear regression and found age is inversely related to mRS at each timepoint (Figure 8K) and CSF Log_2_ Ab titer was directly related to mRS at each time point except at 12 months (Figure 8L). Serum Log_2_ Ab titer had no correlation with mRS at any timepoint.

LOS = length of stay; HSVE = herpes simplex virus meningoencephalitis; NEOS = NMDARE One‐Year Functional Status; mRS = modified Rankin Scale; Individual observations are not displayed in (L) and (M) for clarity, but the full dataset of observed values are available.

## 4 Discussion

This is the largest known single‐center retrospective analysis of pNMDARE, and through this analysis, we found multiple novel and confirmatory observations related to demographics, symptomology, etiologies, workup, and factors affecting outcomes: (1) About half of idiopathic pNMDARE cases present with an exquisitely focal and specific perisylvian phenotype that otherwise resembles a stroke. (2) Two‐thirds of our more than 100 pediatric patients are self‐reported Hispanic (whereas only about one‐third of our diverse city, Houston, TX, USA, are Hispanic), and they have higher biomarkers of CNS inflammation. (3) We corroborate prior reports on mixed‐age and adult cohorts that younger children and those with teratomas have a more severe clinical course and worse outcome in our pediatric‐specific cohort [30 ^,^ 31]. (4) We corroborate prior reports on mixed‐age and adult cohorts that Higher CSF antibody titers at presentation predict worse clinical severity, hospital length of stay, and 12‐month outcomes, independent of the medical interventions themselves [32].

We describe novel initial phenomenology, which appears to approximate to the perisylvian region, that we observed in almost half of our patients and call a “perisylvian phenotype”. Upon examination of the medical records, it became clear that many parents or caregivers described a very specific collection of acute‐onset symptoms in their children as their “day 1 symptoms:” dragging their right leg while walking, complaining of tingling, pain, or numbness on the right side of their body, unable to hold their eating utensils or pencil with their right hand, and unable to get their words out or significantly reduced vocabulary. The cluster of unilateral sensory deficits and weakness with expressive aphasia very commonly initiated an acute stroke workup in the emergency department which was negative for ischemia or hemorrhage on brain imaging, but did note focal perfusion changes on arterial spin labeling (ASL) as we previously described in those who had ASL obtained [33], and diffusion restriction in a minority of patients. The majority of patients initially with perisylvian symptoms had normal conventional brain imaging and some had focal electrographic changes on EEG. We hypothesized that the laterality of perisylvian symptoms matched the hand dominance of the patient, but surprisingly, only two‐thirds of those with perisylvian symptoms lateralized to their motor‐dominant hemisphere. Those with perisylvian symptoms were otherwise no different than the rest of the pNMDARE cohort in terms of biomarkers and long‐term outcomes in our study, but prospective studies are needed to better stratify symptomology by disease course. It is notable that other large studies, particularly those with predominantly adult patients, do not describe this phenomenology in the early days of acute‐onset NMDARE. It is plausible that this phenomenon is a uniquely pediatric phenotype in pNMDARE, perhaps due to the evolving and heterogeneous expression pattern of NR_1_ subunits across various parenchymal foci over the course of childhood development. Alternatively, perhaps the language‐dominant hemisphere is primarily affected, and early expressive aphasia is more susceptible to detection by parents and caregivers, whereas contralateral hemispheric symptoms such as hemineglect may be less sensitive to detection. We propose focal stroke‐like symptoms corresponding to the perisylvian region should prompt a workup of pNMDARE after stroke is ruled out, and our future studies will focus on the proportion of children with stroke‐like symptoms who are ultimately diagnosed with pNMDARE. We define “perisylvian phenotype” by the acute onset of lateralized cortical signs of focal sensory deficit (paresthesia, allodynia, anesthesia) with ipsilateral weakness and expressive aphasia. The phenotype is supported by focal electrographic abnormalities over the temporoparietal region including focal slowing and epileptiform activity and by functional imaging changes such as regional perfusion differences on ASL. We hypothesize there are other imaging and functional abnormalities highlighted specifically in the perisylvian region early in the course of pNMDARE in those with the perisylvian phenotype such as changes seen on fMRI, spectrometry, and 18F‐FDG‐PET in this region.

In addition to perisylvian symptoms, we binned our patients into five other groups including isolated psychiatric symptoms. Consistent with a previous report from a multicenter pNMDARE study including subjects from our institution, those with isolated psychiatric symptoms make up between 5% and 10% of all pNMDARE [3] and these patients were significantly older than patients who presented with other initial symptoms. The median latency from onset of isolated psychosis to the emergence of other conventional pNMDARE symptoms was 3.5 days, which is an important point to consider when developing a differential diagnosis for a child with many *weeks* of psychosis. While teratomas were more associated with isolated psychosis, we cannot say if that is due to increased age, severity of the illness, or a combination of factors.

The demographic distribution of our cohort is interesting: self‐reported Hispanics were disproportionately represented in pNMDARE as the proportion of affected children was 2‐fold greater than the population of our hospital and county. Whether this is due primarily to genetic factors, social determinants of health, or a dynamic combination of both is still to be determined. For example, it may be that Hispanics are predisposed to exposure by a pNMDARE‐triggering infectious agent, thus increasing the odds of pNMDARE. Alternatively, an underlying genetic predisposition to inflammatory conditions may increase the odds of seroconversion to anti‐NR_1_ antibodies after an infectious trigger. It is also possible these two factors function synergistically to affect Hispanics more than the rest of the population. Notably, multiple studies have corroborated our prior report on Hispanic predisposition [29 ^,^ 34 ^–^ 36], and our group has previously reported other encephalitides, such as cortical myelin oligodendrocyte glycoprotein antibody‐associated disorder (MOGAD), affect Hispanics more than non‐Hispanics at our institution [37, 38]. Multi‐center studies are needed, and indeed underway, to investigate the role of socioeconomic drivers of health and outcomes in pNMDARE, taking into consideration the possibility that neighborhood disadvantage and childhood opportunity may be important disparities influencing health outcomes [35].

Cases of pNMDARE occurred most frequently in the summer in our study, with one additional peak in December. Prior studies have reported seasonality in pNMDARE, with separate reports of both children and adults demonstrating higher pNMDARE incidence in the summer. While our findings largely concur with these, our identification of December as a separate peak month has not been previously reported in pNMDARE [39 ^,^ 40]. The seasonality may reflect non‐tumor‐ and non‐HSV‐related infectious triggers such as other Herpesviridae. Most notably, Epstein Barr virus (EBV) may have some degree of seasonality favoring summer months [41] and our group previously suggested EBV may be a more common precursor to pNMDARE than previously thought [29]. Our identification of peak incidence in December demonstrates the need for further studies of this temporality in pNMDARE to identify other potential infectious triggers of the disorder, keeping in mind our cohort was predominantly from the Houston metropolitan area but about one‐third transferred from distinct areas.

Males are equally represented across all age groups while females are more represented in older age groups. Under ten years is split about 50/50 by sex while teenagers with pNMDARE are predominantly female. This may reflect the known impact of puberty and sex hormones on predisposition for autoimmunity [42]. Indeed, females are reported to be disproportionately affected by pNMDARE compared to males which parallels the sexual dimorphism in other autoimmune conditions like multiple sclerosis, which also typically manifests after puberty [43].

In addition to the disproportionate representation by those that self‐report as Hispanic in our cohort, we also found that Hispanics had higher CSF WBC—but not CSF protein, and higher CSF Ab titers—but not serum Ab titers, than non‐Hispanics, despite no differences in latency to presentation, latency to LP, or presenting mRS between self‐reported ethnicity, by language spoken or by ADI. These novel observations may suggest a greater susceptibility of Hispanic children to developing pNMDARE. Higher inflammatory surrogate markers in Hispanic patients and older age may reflect underlying heterogeneity in mounting immune responses by ethnicity and age. Indeed, a recent case series on a mix‐aged French cohort of NMDARE in the ICU found that non‐White patients were more likely to develop medically refractory NMDARE, albeit their demographic makeup is likely vastly different from ours [31]. When accounting for confounders that may drive health outcomes, we found no such difference in surrogates of CNS inflammation (CSF WBC and Ab titers) by socioeconomic disadvantage (ADI). However, ADI is merely one of many quantitative metrics used to study social determinants of health, and the lack of statistical difference in our cohort does not indicate the absence of social determinants of health, but it may suggest that income and education levels are not the *primary* drivers. Future studies will investigate how—and if—ethnicity drives these biomarkers of disease, or rather, if ethnicity is merely a confounding variable driven itself by underlying societal structural factors and access barriers. Likewise, our ongoing studies are evaluating potential genetic risk alleles that may confer either increased odds of developing an autoimmune condition or drive biomarkers of CNS inflammation, and if these alleles are enriched in certain populations.

When we compared CSF WBC by those who had an earlier lumbar puncture (the first ten days of symptom onset) to those who had it later, we observed a significantly higher cell count in those in the earlier group, and importantly, this observation was independent of age. Conversely, age was *inversely* correlated to serum Ab titer but there was no correlation with CSF Ab titer, which suggests there may be age‐dependent immune mechanisms that alter the amplitude of antibody production in the periphery. Patients with teratomas presented the earliest, had the earliest LPs, and had the highest CSF WBC, although we were unable to detect a difference in Ab titer, much like had been previously reported [32], due to low sample size in the teratoma group. We propose this is because those with teratoma‐associated pNMDARE have the quickest progression to serious symptoms and the most severe symptoms at their peak of illness. It is not possible to measure the symptom progression prior to hospital presentation or throughout the subsequent years with the existing dataset, however future studies will focus on family surveys to quantify and track symptom severity over time.

Our study identified CSF Ab titers as among the most important factors in outcomes, including a direct correlation to hospital LOS and mRS at presentation through 12 months, in relative concordance with prior studies [32]. Notably, latency from symptom onset to presentation within seven days was associated with significantly longer LOS than later presentations. We did not detect significant differences in LOS or outcomes based on treatment given or latency to treatments, however most of our patients received the same cocktail of interventions (high dose IV steroids, IVIG, and rituximab x2 ± PLEX), which makes the statistical comparison among treatment options nearly impossible. Based on our analysis, CSF Ab titer may therefore have superior predictive value in outcomes. We did not test serial Ab titers in this study, but future research may focus on how Ab titer curves may affect clinical severity and outcomes.

Excluding those with HSVE‐associated pNMDARE, most children had an unremarkable initial brain MRI and this was independent of the latency to obtaining the MRI, sex, ethnicity, age, initial symptomology, or Ab titers. It is critical for clinicians to remember that there is no imaging signature of pNMDARE, but rather, imaging is important to rule out other causes of symptoms such as stroke. Likewise, half of our children with pNMDARE had an unremarkable initial EEG which was independent of latency to obtaining the EEG or demographic characteristics described above. We did detect a statistically significant difference in presenting mRS based on initial EEG result, but the clinical significance of this is unknown and may be marginal. The abnormal EEGs were composed of focal discharges, focal slowing, seizures, and in a minority of cases beta activity superimposed over delta activity (delta‐brush). CSF WBC was normal in more than a quarter of children with idiopathic pNMDARE, indicating a lack of sensitivity for the presence of central leukocytes. Taken together, imaging and EEG are important tools to identify *mimickers* of pNMDARE during the early workup period, but there are no sufficiently sensitive or specific imaging or electrographic signatures of pNMDARE itself. Further, when considering autoimmune encephalitis in a differential diagnosis, the lack of CSF WBC is not an absolute rule out. One of the most clinically impactful corroborating findings of our study is that normal CSF WBC or a normal MRI of the brain significantly delayed initiation of first‐line immunotherapy. In our practical experience, normal routine CSF studies is a frequent rationale for providers to pivot their management toward an alternative diagnosis until the emergence of “classical NMDARE symptoms” (i.e., a combination of choreiform movements, seizures, and/or psychosis). We suggest that if a pediatric patient has any of the described initial phenotypes, their healthcare team continues to entertain pNMDARE until CSF or serum Ab testing proves otherwise.

Finally, there was an interestingly high proportion of patients with mTBI preceding the onset of pNMDARE symptoms that requires further consideration. Eight of our patients experienced mTBI approximately two weeks prior to the onset of their pNMDARE symptoms with a narrow range of observed latencies and *no* interim symptoms. Our initial impression of these cases was that the patients were likely experiencing early symptoms of pNMDARE which caused their fall and head trauma. But considering the rapid progression of pNMDARE symptoms, we felt the odds of pNMDARE precipitating the trauma two weeks prior to any other symptom were low. We next considered recall bias to play a considerable role in the caregiver's history upon presentation with pNMDARE symptoms. However, the mTBIs described to clinicians were quite notable clinical events, and four subjects even presented to the ER immediately after the mTBI indicating the trauma was not merely a marginal everyday event. None of the eight subjects with preceding mTBI had any other identified precipitant of pNMDARE (teratoma or HSVE), which further increased the odds that head trauma could be responsible. We are unsure if head trauma was merely coincident or a precipitant of pNMDARE, but given the remarkably similar latencies to symptom onset, the absence of other recognized etiologies, and the overall reluctance to report unconventional correlations in the literature, we felt compelled to report this novel observation in pNMDARE to open the dialogue among neurologists who may have their own similar cases to report. There are any number of potential biological mechanisms we may be able to rationalize for mTBI as a pNMDARE etiology, including secondary injury following the trauma, interruption of parenchymal immune privilege, and even as a protective measure against glutamate excitotoxicity. Future studies are needed to investigate these possibilities, but most importantly for now, our goal is to share this novel clinical observation from a large volume academic center and ask if other clinicians have hypothesized a similar etiology that may partially tap into the elusive idiopathic group.

There are several limitations to our study. Chiefly, we performed a retrospective chart review spanning several years, which includes the first patient diagnosed with pNMDARE at our institution in 2009 to recently diagnosed patients in 2024. In addition to individual practice variations, our cohort includes changes in practice patterns and disease awareness after the disease was first published in 2007 [44]. However, we protocolized our approach to diagnosing and treating children with suspected autoimmune encephalitis with a focus on early rituximab in 2009 [45], leading to more consistent practice patterns than most institutions. Additionally, some of our statistical analysis was performed post hoc, which risks spurious findings but allows for hypothesis generation. Our institution is a large tertiary/quaternary referral center in the fifth‐most populous Metropolitan Statistical Area in the country [19], which introduces referral bias that may skew toward severe clinical courses and worse outcomes. Indeed, about one‐quarter of our cohort was worked up and managed elsewhere prior to transfer to our hospital for ongoing management. Still, we did not detect any difference in biomarkers of CNS inflammation (CSF WBC or Ab titer) or mRS over time when comparing those who were worked up at our hospital vs. those managed elsewhere prior to transfer to us. Our ongoing studies aim to characterize demographic trends across the state of Texas. Additionally, there are no perfect metrics of long‐term outcomes in pNMDARE; while NEOS is a validated tool to predict functional recovery, its implementation in pediatric cohorts is limited. Strengths of our study include the large sample size of children from one center with standardized protocols, allowing for greater replicability and generalizability across the pNMDARE international population. Additionally, the diversity of our region allowed for greater stratification of cases based on multiple reliable factors, including by ethnicity, which permitted reasonable conclusions on the predisposition of Hispanic children for pNMDARE. Importantly, we hope our novel description of a perisylvian phenotype as an early defining feature of idiopathic pNMDARE comes to the attention of pediatricians and general neurologists everywhere so they too can identify, workup, and manage pNMDARE early in the disease course to improve long‐term outcomes.

## Funding

Through support from the Research Vision at Texas Children's Hospital.

## Conflicts of Interest

The authors have declared that no conflicts of interest exists.

## Supporting information




**Supporting File**: advs75029‐sup‐0001‐FigureS1.docx.

## Data Availability

Anonymized data not published within this article will be made available by request from any qualified investigator.
