# Supplementary material for: Phenotypic Diversity and Outcomes in Pediatric NMDA Receptor Encephalitis: A 15‐Year Retrospective Study from the Largest Children's Hospital in the United States
Source: Adv Sci (Weinh). 2026 Mar 28;13(28):e20313. doi: 10.1002/advs.202520313 (PMC13185876; doi:10.1002/advs.202520313)
Supplement: Supplementary file 1 — Supporting File: advs75029‐sup‐0001‐FigureS1.docx. [file ADVS-13-e20313-s001.docx]

**Supplemental Figure.** **(A)** 77/115 patients (67%) speak English or are bilingual with English while most of the remaining patients speak Spanish only. **(B)** 2 patients did not have ADI available. 23/113 patients (20.3%) had an ADI of 1-3, 58/113 patients (51.3%) had an ADI of 4-7, and 32/113 patients (28.3%) had an ADI of 8-10. **(C)** ADI was significantly lower (less socioeconomic disadvantage) in patients who self-reported non-Hispanic compared to Hispanic (2.6 +/- 0.44 vs 6.4 +/- 0.29, p<0.01). **(D)** After applying exclusion criteria, latency to presentation did not differ between language spoken (English or Bilingual: 3.6 +/- 0.8 days vs Non-English Only: 5.0 +/- 1.1 days, p=0.14), **(E)** nor did it differ by ADI (1-3: 6.0 +/- 1.5 days vs 4-7: 3.6 +/- 0.9 days vs 8-10: 2.2 +/- 0.5 days). **(F)** CSF WBC did not differ by ADI (1-3: 30.1 +/- 11.8 cells vs 4-7: 25.6 +/- 5.2 cells vs 8-10: 26.1 +/- 7.0 cells). **(G)** CSF WBC did not differ between those who were worked up and managed at our institution vs those who were managed elsewhere before transfer. Log_2_ Ab titer did not differ between any **(H)** ADI group or **(I)** by transfer status in either CSF or serum. ADI = Area Deprivation Index; mRS = modified Rankin Scale

**
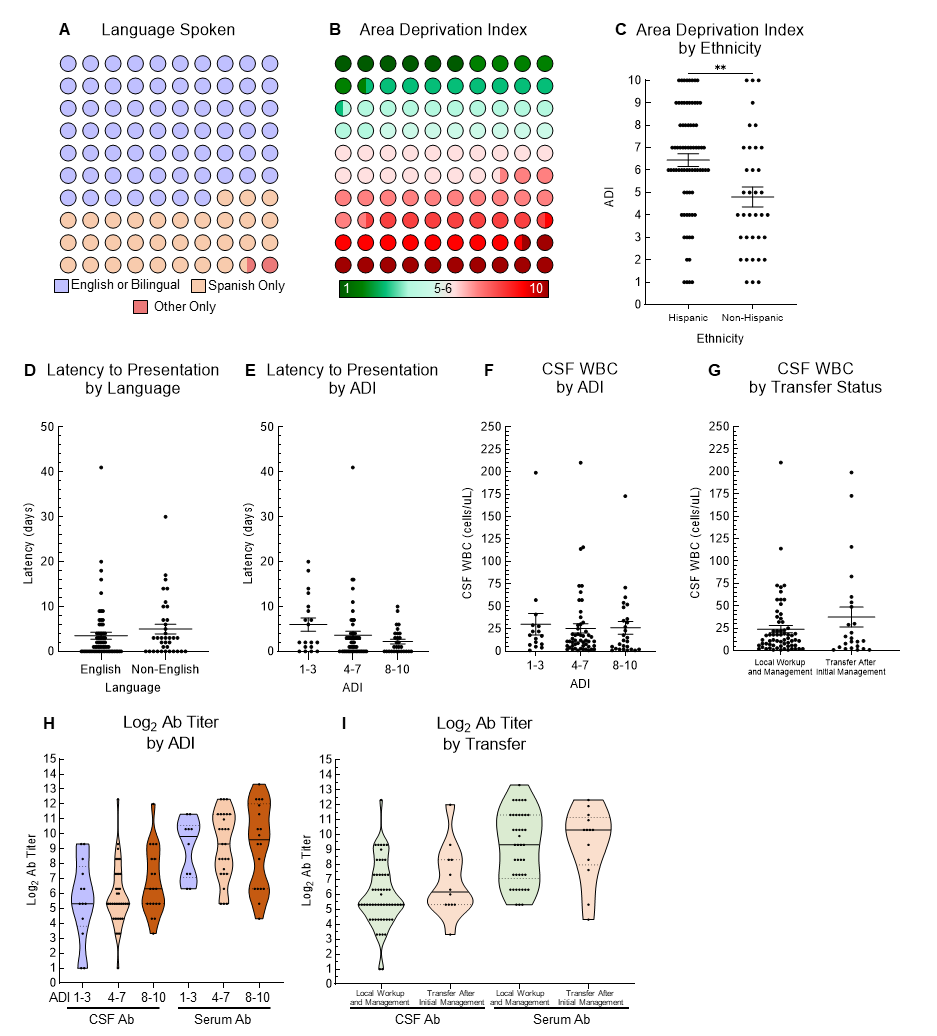
**
